# Cognitive Performance in Asymptomatic Elderly People with Hepatitis C: The Role of Education

**DOI:** 10.3390/jcm12144588

**Published:** 2023-07-10

**Authors:** Max Kopti Fakoury, Sergio Luis Schmidt, Carlos Eduardo Brandão Mello, Aureo do Carmo Filho, Marcia Maria Amendola Pires

**Affiliations:** 1 Department of Internal Medicine, Gaffrée and Guinle University Hospital, Federal University of the State of Rio de Janeiro, Rio de Janeiro 21941-853, Brazil; cedubrandao@gmail.com; 2 Graduate Program in Neurology, Department of Neurology, PPGNEURO-UNIRIO, Federal University of the State of Rio de Janeiro, Rio de Janeiro 21941-853, Brazil; slschmidt@terra.com.br (S.L.S.); aureocf@gmail.com (A.d.C.F.); mmamendola@gmail.com (M.M.A.P.); 3 Laboratory of Behavioral Neurology, Federal University of the State of Rio de Janeiro, Rio de Janeiro 21941-853, Brazil; 4 RENASCER Multidisciplinary Program of the Third Age, Gaffrée and Guinle University Hospital, Federal University of the State of Rio de Janeiro, Rio de Janeiro 21941-853, Brazil; 5 Graduate Program in HIV/AIDS Infection and Viral Hepatitis, PPGHIV/HV-UNIRIO, School of Medicine and Surgery, Federal University of the State of Rio de Janeiro, Rio de Janeiro 21941-853, Brazil

**Keywords:** chronic hepatitis C, elderly, viral hepatitis, cognitive reserve, mental status tests, education, literacy, clock drawing test

## Abstract

Background: Neurotropism of the hepatitis C virus (HCV) can be the source of subtle neuropsychological symptoms in non-cirrhotic patients. Age is a risk factor for cognitive impairment (CI). Thus, asymptomatic elderly people who carry HCV might be at a greater risk of CI. Education can influence test performance. Objectives: (1) To verify whether elderly people with HCV performed poorer than controls on cognitive tests. (2) To analyze how education affects performance. (3) To verify whether the extent of the effect of education on performance depends on the group (HCV vs. controls) and the type of cognitive test. Methods: Asymptomatic HCV carriers older than 60 years (*n* = 41) were matched with 41 corresponding controls. All participants performed the following tests: Mini-Cog, Mini Mental State Examination, clock drawing test (CDT), and verbal fluency. Results: (1) There were no significant differences in cognitive performance between the two groups. (2) Higher education was always associated with better performance. (3) There was a significant group difference in the slopes of the regression lines between years of education and CDT performance. No differences were found for the other three tests. Conclusion: Considering the scores on the CDT, the rate of improvement in performance when schooling increases is higher in HCV carriers.

## 1. Introduction

According to the World Health Organization, more than 70 million people worldwide have the hepatitis C virus (HCV) [1]. Approximately 40% of people infected with HCV are elderly (i.e., age > 60 years) [2]. Progression to cirrhosis and, eventually, to hepatocellular carcinoma occurs more frequently in patients who acquire the infection in old age [3,4]. However, most HCV-infected elderly people acquired HCV when they were young, and the disease is asymptomatic with a benign course [2]. Despite the high number of asymptomatic elderly people with HCV, previous studies have frequently included patients with clinically detectable symptoms or those who are referred to hospitals. Since 2018, Brazilian public health authorities have promoted a complimentary treatment for HCV with direct-acting antivirals (DAAs), which has included clinically and laboratory asymptomatic HCV-infected individuals [5]. These asymptomatic HCV carriers do not exhibit clinical complaints and usually have unremarkable laboratory exams. Most of these asymptomatic individuals were infected with HCV after receiving blood transfusions in the early 1980s. It is estimated that 650,000 Brazilians have HCV, and half of these individuals are elderly [5,6]. The present study was conducted in Brazil and included only asymptomatic elderly people with HCV. Here, they will be referred to as HCV carriers, or simply as the HCV group.

Despite liver involvement, HCV infection is considered to be a systemic infection due to the ability of the virus to penetrate and replicate in the main organ systems, including the central nervous system (CNS) [7]. Several mechanisms have been proposed regarding the neurotropism of HCV, such as direct viral damage and immune-mediated vascular damage [8,9,10,11]. Accordingly, previous studies have described cognitive changes unrelated to advanced liver disease and, therefore, distinct from complications observed in patients with hepatic encephalopathy or cirrhosis [8,9]. Furthermore, in HCV patients without cirrhosis, magnetic resonance spectroscopy associated with perfusion-weighted imaging and diffusion tensor imaging has shown white-matter neuronal loss, alterations of association tracts, and cortical hypoperfusion [12]. Thus, the presence of HCV in the brain may be a direct pathway for neurocognitive impairment, independent of the severity of liver involvement [13]. However, few data are available on neurocognitive performance in asymptomatic elderly people who had been infected with HCV when they were younger. This is particularly important because HCV carriers are considered to be the main reservoir of the virus [4,14]. However, it is unknown to what extent the neurotropism of HCV exacerbates cognitive impairment in the elderly, or whether it triggers progressive neurodegenerative brain damage.

Several risk factors have been found to be associated with cognitive decline in older adults, such as low education, aging, history of traumatic brain injury with known loss of consciousness ≥30 min, alcoholism, depression, and the use of illicit substances or medications with cognitive effects [15,16,17,18,19,20]. Another variable is sex, because some studies have suggested that women have a higher risk of Alzheimer’s disease [17,19]. In the present study, we considered all of the abovementioned risk factors.

Among the factors that interfere with cognitive performance, the number of years of formal education deserves particular emphasis. Several cognitive tests, such as the Mini Mental State Examination (MMSE) and verbal fluency tests (VFTs), are strongly associated with years of formal education. Indeed, the vocabulary and arithmetic skills present in some neuropsychological tests depend heavily on formal education. One way to circumvent this problem and reduce variability is the use of norms based on years of formal education. However, research has shown that years of education may overpredict estimated reading and mathematical levels in Latin Americans [21]. In Brazil, the association between years and quality of education depends on demographic variables such as location of schooling, place of birth, and parental literacy [22]. Moreover, it is expected that more than 50% of Brazilians read at least 3 or more years below their educational level [21,22]. Therefore, the use of Brazilian norms based on years of formal educational is controversial. To circumvent these limitations, the participants of the control group of this study were attending the same hospital where all of the HCV carriers were selected.

Another important issue regarding the effect of education on test performance is related to cognitive reserve. Higher levels of cognitive reserve can be protective against the neuropsychological manifestation of neural injury across a variety of clinical disorders [20,23,24]. Cognitive reserve is characterized by life experiences, in combination or interaction with genetic factors, explaining the individual’s susceptibility to deal with brain diseases and aging [23]. As it cannot be observed directly, it is measured using surrogate variables, including educational and occupational achievement, lifestyle with cognitively stimulating activities, socioeconomic status, and early life experiences [24,25,26]. The most frequently used variable is education level, which is associated with a lower risk of mild cognitive impairment and dementia. However, as mentioned in the above paragraph, the valid use of years of education in neuropsychological research is only admissible when the quality of education of the groups that are being compared, is equivalent.

Specifically, in our sample, the education level was used as a proxy for cognitive reserve because it adequately captured the quality of learning based on the subjects’ residence and the year when they completed their formal education [16,20,27,28]. Although individuals with different levels of cognitive reserve may accumulate the same number of neuropathological burdens during aging, those with greater cognitive reserve would not have detectable symptoms of cognitive impairment even though they already had neuropathological changes [16]. However, the role of cognitive reserve in neurocognitive deficits among asymptomatic elderly people who carry HCV is not well understood.

In the present study, we formulated three hypotheses: Firstly, we hypothesized that the general cognitive performance of HCV carriers would be poorer than that of controls after controlling for age, sex, and education, as described for other infectious diseases [29,30,31,32]. Secondly, we considered the possibility that higher-educated subjects would perform better than low-educated subjects. Thirdly, we also considered the possibility that education could interact with performance in specific tests and obscure potential differences between controls and HCV carriers.

### Objectives

The first main objective of this study was to investigate whether elderly people who carried HCV showed a poorer cognitive performance than non-infected elderly subjects, after controlling for sex, age, comorbidities, depression, lifestyle, and years of formal education. The second main objective was to analyze how education affected performance. Thirdly, we verified whether there were group differences (controls vs. HCV carriers) in the strength of the association between years of education and cognitive performance that could obscure the expected average score differences in specific tests. A secondary objective was to investigate whether the viral load in HCV carriers was associated with cognitive performance, sex, or age.

## 2. Methods

### 2.1. Participants

HCV group: Data were collected from HCV-infected patients (aged 60 years or older) prior to DAA treatment from May 2018 to February 2020 at the Gaffrée and Guinle University Hospital, which is accredited by the Brazilian Ministry of Health as a treatment center for HCV.

Diagnosis of hepatitis C: Anti-HCV reactive and HCV RNA detectable for more than six months.

The patients underwent a global health assessment, which provided information on their epidemiological and comorbidity profiles, functionality, and general wellbeing. We collected data on liver function, degree of fibrosis, viral load, and genotype.

Control group: The data for the control group were collected in the same evaluation period as those for the elderly patients with HCV. We included elderly participants of the third-age program in the same hospital as the HCV patients. The program (named “Renascer”, which means “to be reborn”) has been running since 1995 at the Gaffrée and Guinle University Hospital in Rio de Janeiro, Brazil. The program aims to minimize social isolation and promote elderly people’s enjoyment and wellbeing. Membership is open to anyone aged 60+ years and retired or no longer working full time and interested in participating in social, educational, physical, and leisure activities. The program promotes courses and social activities for older persons, irrespective of background. Elderly people enrolled in the program receive specific health screenings, including routine blood and physical exams. Additionally, it should be stressed that the control group did not include inpatients or outpatients. Therefore, all of the subjects in the control group were in good health. In this group, the same procedures for global assessment and cognitive screening tests were used.

Inclusion criteria: Sixty years of age or older, without functional complaints, and with no changes in the basic and instrumental activities of daily life.

Exclusion criteria: Participants with the hepatitis B virus or HIV, previous diagnosis of major cognitive disorder (based on medical files), use of medications known to interfere with cognitive functions (e.g., illicit drugs, antipsychotics, antiepileptics, benzodiazepines, and alcohol abuse), and the presence of decompensated chronic non-communicable diseases (e.g., arterial hypertension, diabetes mellitus, hypothyroidism, diagnosis of dementia syndrome, and depression).

### 2.2. Procedures

The sociodemographic and educational characteristics, as well as the clinical and evolution data of the comorbidities, were obtained by analysis and review of medical records at the first appointment during the study period.

Education was analyzed as a continuous variable (years of formal education) and also as a binary variable (low and high levels of education, where low indicates <8 years of formal education).

Functional scales of basic and instrumental activities of daily living were also applied and recorded. In both of the two groups, depression symptoms were screened using the Hamilton Depression Scale and the Geriatric Depression Scale.

In the HCV-infected group, the degree of fibrosis was assessed using noninvasive tests (FIBROSCAN^®^ transient liver elastography). According to the Metavir score for fibrosis grading, individuals were categorized as follows: F0 (absence of fibrosis), F1 (minimal fibrosis), F2 (septal fibrosis), F3 (various portal-portal septal fibrosis or fibrosis without cirrhosis), and F4 (fibrosis–cirrhosis) [33].

All participants underwent blood tests to evaluate their red blood cell, leukocyte, and platelet counts, changes in liver enzymes (AST, ALT, and GT-gamma), and liver function (prothrombin time, albumin, and bilirubin), all of which are important tests for the Child–Pugh classification of patients infected with HCV. In the control group, we only included participants with normal results in all of these tests.

The HCV genotyping was performed by sequencing, to discriminate between genotype 1 and non-genotype 1 (genotypes 2–6). Quantitative HCV RNA viral load was measured by RT–PCR (lower limit of detection: 12 IU/mL) (Cobas Monitor Roche) at baseline.

Evaluation with cognitive screening tests was performed by physicians trained in the application of these instruments in an appropriate and safe environment, and all of the tests were translated and validated for the Portuguese language.

Tests applied:

- **Mini-Cog**: In this test, the patient was asked to memorize and repeat three unrelated words. After that, they were asked to draw a clock face and place the numbers in the correct positions. Then, the patients were asked to draw the hands to mark 11 h and 10 min. Here, the participants drew their own clock circumference, and this subtest was scored using a 2-point scale, with higher numbers indicating better performance. Then, the patients were asked to repeat the three words that were initially given to them. Total scores ranged from 0 to 5 according to the performance of the patient [15].
- **Mini Mental State Examination (MMSE)**: The translation to the Brazilian Portuguese language was proposed by Bertolucci et al. [34] and adapted by Lourenço and Veras [35]. The score ranged from 0 to 30 points. The MMSE consists of 11 items, with a maximum score of 30. The first part of the test (items 1 to 5) mostly assesses memory and executive function (attention and concentration). The second part (items 6 to 11) also assesses other cortical functions (e.g., language, gnosis, praxis, executive function, and visuospatial function). The three unrelated words of the MMSE differed from those previously used in the Mini-Cog test.
- **Spreen–Benton verbal fluency test (VFT)**: This test was validated in Brazil by Brucki et al. [36]. In the present study, we only used the subtest that involves naming animals in one minute. This evaluates the ability to evoke words from semantic categories under directed conditions, and the score corresponds to the total number of animal names produced in one minute (executive function). We asked the patient to speak as many words as possible, in one minute, in the animal category. All animals were counted, except for repetitions and regular oppositions of sex (e.g., cat/cat = 1 point; ox/cow = 2 points). The final score is the total number of valid words in 1 min.
- **Clock drawing test (CDT)**: This test consists of drawing a clock face without numbers. Next, participants are asked to add the numbers in the correct positions, and then the hands of the clock representing a specific time (8 h and 20 min). The test includes a pre-drawn circle 10 cm in diameter. The subjects were instructed to draw numbers within this pre-drawn circle to make that circle look like the face of a clock. After completion, the clock face was divided into quadrants, and the scoring system yielded an overall 10-point scale, with higher numbers indicating better performance.

There is currently no suitable neuropsychological assessment to effectively identify early cognitive impairment in elderly people. The most widely used screening test for cognitive impairment is the Mini Mental State Examination (MMSE) [37]. However, in China, the Mini-Cog was found to be superior to the MMSE in identifying mild cognitive impairment because it is less affected by age and education level [38]. The Mini-Cog consists of a free clock drawing test and a three-item recall. The clock drawing test (CDT) has a known potential for detecting cognitive impairment in populations with mild cognitive impairment, and the clinical use of the CDT has increased considerably [39]. In addition, the clinical utility of different CDT scoring systems has shown significant differences in detecting cognitive deficits in several pathologies [39]. Finally, verbal fluency—particularly semantic fluency—has been found to be an accurate and efficient tool in screening for early cognitive impairment [40,41]. Therefore, these four instruments were considered to be reliable tools for a short and quick initial screening of mild cognitive deterioration.

### 2.3. Statistical Analysis

*t*-tests for independent samples (for continuous variables) and chi-squared tests (for non-continuous variables) were used for examining group differences across demographic and clinical variables.

Two groups of elderly people were compared: HCV carriers vs. controls without HCV. All of the statistical procedures were performed using the results of the cognitive screening tests as dependent variables and the following cofactors: sex (male and female), age (60 to 69 years, 70 to 79 years, and 80 years or older), and years of formal education. We also performed all statistical analyses considering education as a binary variable (low and high, where low indicates <8 years of formal education).

We transformed all raw scores of the 4 tests into Z-scores using the means and the standard deviations of the control group. The use of the standardized scores allowed direct comparisons across the 4 tests.

To achieve the 1st main objective, a MANCOVA (multivariate analysis of covariance) was performed to examine the differences between groups (HCV carriers vs. controls) in the Z-scores of the cognitive screening tests, using age, sex, and education as cofactors. Box’s M test was used to evaluate the homogeneity of the covariance matrices. Box’s M test was interpreted together with the inspection of logarithmic determinants [42]. If the assumption of sphericity was violated, Greenhouse–Geisser correction was applied. Univariate ANCOVAs were subsequently performed to analyze group differences for each test. For the MANCOVA and for each one of the univariate ANCOVAs, η^2^ (eta squared) was used to calculate the effect size of the results. Cohen suggested that η^2^ = 0.01 should be considered a “small” effect size (η^2^), 0.06 represents a “medium” effect size, and 0.14 a “large” effect size. We performed these tests considering education as a continuous variable. We also performed a confirmatory analysis using educational level as a binary variable (low and high, where low indicates <8 years of formal education). Bonferroni correction was applied for multiple comparisons.

As MANCOVAs and ANCOVAs only compare means, we also studied possible differences between HCV and controls considering the entire distribution for each test. The Kolmogorov–Smirnov (2 samples) test was used because it is sensitive to any differences in median, dispersion, and skewness, enabling the study of the difference between the two entire distributions (HCV vs. controls, for each test).

To achieve the 2nd main objective, a MANCOVA (multivariate analysis of covariance) was performed to examine the differences between the two educational levels (high vs. low) in the Z-scores of the cognitive screening tests, using age and sex as confounders. Box’s M test inspection of logarithmic determinants and Greenhouse–Geisser correction were also applied. Univariate ANCOVAs were subsequently performed to analyze group differences for each cognitive test. We also performed this analysis separately for each group (HCV and controls). As described for the first objective, the effect sizes of the results were estimated by η^2^.

To achieve the 3rd main objective, we performed a repeated within-subject design considering the Z-scores as the repeated within-subject variables, since the same subjects performed the 4 tests. Then, a repeated-measures MANOVA was performed on the Z-scores. Within-subject factor: cognitive tests (4 levels). Between-subject factor: group (HCV carriers and controls). Years of formal education was used as a continuous predictive variable. Therefore, the triple interaction (group x cognitive test x education) was analyzed. If the assumption of sphericity was violated, Greenhouse–Geisser correction was applied. The effect size of each result was estimated by η^2^ (eta squared). We also performed a confirmatory statistical analysis considering education as a binary variable (low and high, where low indicates < 8 years of formal education).

In the present study, we balanced the groups considering potential confounders. We included the confounders to account for the variance that they explained in the dependent variables. As pointed out by previous investigations [43], we considered that matching is more complex than just balancing groups for potential confounders. Therefore, our matched design controlled for all important matching factors in the analysis (i.e., age and sex).

However, the inclusion of confounders has a negative impact on statistical power. Although some investigators have proposed that the benefit obtained from accounting for the variance explained by the confounders in the dependent variables is greater than the increase in power obtained without confounders [43], it is also possible to consider that matching can break the association between confounders and the variables of interest. Thus, as we used a matching design, it was possible to perform the MANOVAs and corresponding ANOVAs without confounders (age, sex, and education). Considering that this approach could increase the statistical power, we also performed these tests without confounders (first and third objectives).

For each separate group, the relationship between years of education and the dependent variables (cognitive performance on each of the 4 tests) was analyzed with the aid of Pearson’s correlation coefficients. Group differences in the slopes of the regression lines were analyzed by computing the difference between the two slopes divided by the standard error of the difference between the slopes.

To achieve the secondary objective, we performed a correlational analysis in the group of HCV carriers. We measured the strength and direction of the relationships between viral load and each of the following variables: educational level, scores on each cognitive test, sex, and age. Depending on the type of variable (continuous, categorical, or binary), we performed point-biserial correlation or calculated Pearson’s correlation coefficients.

When necessary, correction for multiple comparisons was performed with the aid of Bonferroni corrections to keep the type I error at 5% overall. For all statistical procedures, significance was set at 5% (one-tailed).

### 2.4. Ethical Considerations

The study followed the guidelines of the Declaration of Helsinki, was approved by the Ethics Committee of the Gaffrée and Guinle University Hospital under code CAAE:12630419.0.0000.5258, and was evaluated and approved under opinion number 3358.238. All participants were oriented and informed about the purposes of using their clinical data for research through the Free and Informed Consent Form. All who agreed to participate in the study signed the informed consent form. Only two participants (one in each group) did not authorize the use of their data.

## 3. Results

### 3.1. Demographics and Clinical Date

During the study period, 49 HCV patients were initially included. After applying the exclusion criteria, eight participants were excluded. The main characteristics (clinical, epidemiological, and virological) related to HCV infection are shown in Table 1, including viral load, route of transmission, genotype of HCV, Child–Pugh scale, degree of fibrosis, date (year) in which the patient was exposed to HCV, and the year of the serological diagnosis.

In the evaluation and selection of the control group, we initially had 54 participants. After applying the exclusion criteria and pairing with the HCV group, we selected 41 participants. All of them had laboratory (blood) tests within the normal range.

Demographic and clinical variables, along with the respective comparisons between the two groups, are shown in Table 2.

### 3.2. Analyses of Group Differences in Cognitive Performance, Controlling for Age, Sex, and Education (First Objective)

The raw scores of the cognitive tests are shown in Table 3.

We examined the performance in the four cognitive tests, using Z-scores as dependent variables, group as the independent variable, and sex, age, and years of education as covariates. The cognitive performance did not differ between the two groups (F = 1.25, df = 4/74, *p* = 0.15, η^2^ = 0.06); education affected performance in both groups (F = 4.40, df = 4/74, *p* < 0.01, η^2^ = 0.20), but sex and age did not reach significance. After excluding age and sex, a follow-up MANCOVA examined the associations between the dependent variables and group, using only years of educational level as a cofactor. We still found a nonsignificant multivariate effect of the participant’s group (F = 1.54, df = 4/76, *p* = 0.10, η^2^ = 0.08) and a significant effect of educational level (F = 4.51, df = 4/76, *p* < 0.01, η^2^ = 0.19).

A confirmatory MANCOVA also verified the effect of group on test performance, considering level of education (high and low) as a cofactor. Sex and age were also used as covariates. The cognitive performance did not differ between the two groups (F = 1.40, df = 4/74, *p* = 0.13, η^2^ = 0.07). Similarly, sex and age did not reach significance. Conversely, education levels affected performance in both groups (F = 4.40, df = 4/74, *p* < 0.01, η^2^ = 0.19). After excluding age and sex, a follow-up MANCOVA examined associations between the dependent variables and group, using only educational level as cofactor. We still found a nonsignificant multivariate effect of group (F = 1.76, df = 4/76, *p* = 0.08, η^2^ = 0.08), and educational level remained significant (F = 4.52, df = 4/76, *p* < 0.01, η^2^ = 0.19).

As explained in the Methods section, we also performed MANOVAs and ANOVAs without any confounders (age, sex, and education). The results did not change, confirming the absence of average differences in performance between the two groups. In addition, the Kolmogorov–Smirnov (2 samples) test did not detect any group differences in the median, dispersion, or skewness for any of the four cognitive tests. Thus, the analyses based on the Kolmogorov–Smirnov (2 samples) tests indicated that the distributions of the cognitive scores were the same across the categories of the group (HCV and controls).

Considering the results, we rejected the hypothesis that cognitive performance was lower in HCV carriers as compared to paired controls.

### 3.3. Educational Effect (Second Objective)

We performed a MANCOVA to test the effect of education on test performance, considering levels of education (high and low) as the independent variable. Initially, sex and age were used as covariates. As expected, education levels affected performance (F = 4.56, df = 4/75, *p* < 0.01, η^2^ = 0.20). Univariate ANCOVAs indicated a poorer performance in the lower-educated group on three tests (MMSE, VFT, and CDT). Conversely, education level did not affect performance on the Mini-Cog. After excluding the confounders (age and sex), the MANOVA confirmed the effect of education on performance (F = 4.73, df = 4/77, *p* < 0.01, η^2^ = 0.20).

When the two groups were analyzed separately, the ANCOVAs showed that educational level remained significant in three tests in the HCV group (MMSE, VFT, and CDT). In the control group, however, the educational level reached significance in only two tests (MMSE and VFT). In both groups, the effect of educational level on Mini-Cog performance was not significant. It should be mentioned that as each group was analyzed separately, we always considered the two covariates (age and sex). In the control group, the effect of educational level on CDT performance did not reach significance (F = 0.18, df = 1/37, *p* = 0.33, η^2^ = 0.005), whereas in the HCV group, CDT performance depended on educational level (F = 3.04, df = 1/37, *p* = 0.045, η^2^ = 0.08).

**Figure 1:**
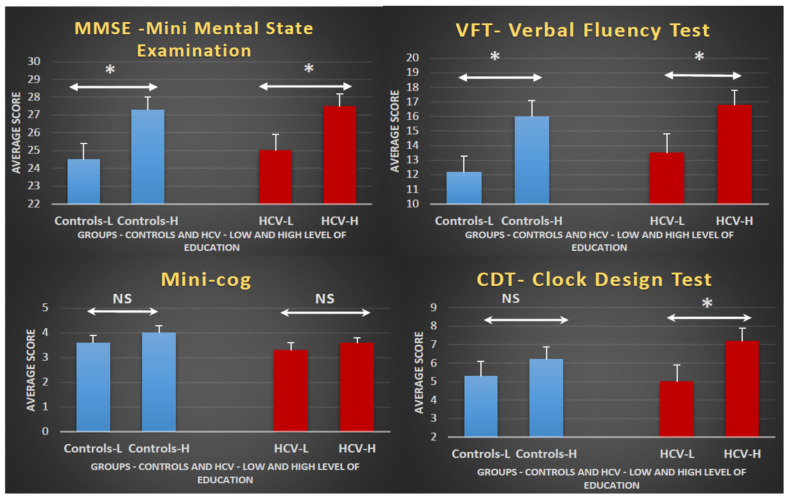
Effect of education level on cognitive performance in controls and HCV carriers: Bar graphs displaying the mean ± standard error per group (controls or HCV), separated by educational level (high or low) for each test. The diagrams indicate that subjects with a higher education level (H) always perform better than those with a lower education level (L), irrespective of the group (controls or HCV). The statistical analyses show that differences reach significance in the MMSE and VFT independent of the subjects’ group. Although the performances in the Mini-Cog are better in higher-educated participants in both groups, the differences are not statistically significant. For the CDT, the effect of education on performance only reaches statistical significance in the HCV group. HCV, hepatitis C virus; * *p*-value < 0.05; NS, non-significant; low education indicates <8 years of formal education; high education indicates ≥8 years of formal education.

In general, a higher educational level was associated with a better performance (Figure 1). In both groups, the quantitative analysis indicated that performance on the MMSE and VFT was significantly affected by education level, but this did not reach significance on the Mini-Cog. Conversely the effect of education level on the CDT reached significance only in the HCV group.

### 3.4. Analyses of Group Differences in the Effect of Educational Level on Specific Tests (Third Objective)

The repeated MANOVA for the Z-scores indicated a statistical significance for the interaction between group, type of test, and years of education (F = 1.90, df = 6/154, *p* = 0.04, η^2^ = 0.07). The interaction between group and years of education was significant (F = 5.25, df = 2/79, *p* < 0.01, η^2^ = 0.12).

When we performed the repeated MANOVA for the Z-scores replacing years of education with level of education (high and low), we still found a significant interaction between group, type of test, and level of education (F = 1.88, df = 6/154, *p* = 0.04, η^2^ = 0.07). The interaction between group and years of education was significant (F = 4.82, df = 2/79, *p* < 0.01, η^2^ = 0.11).

**Figure 2:**
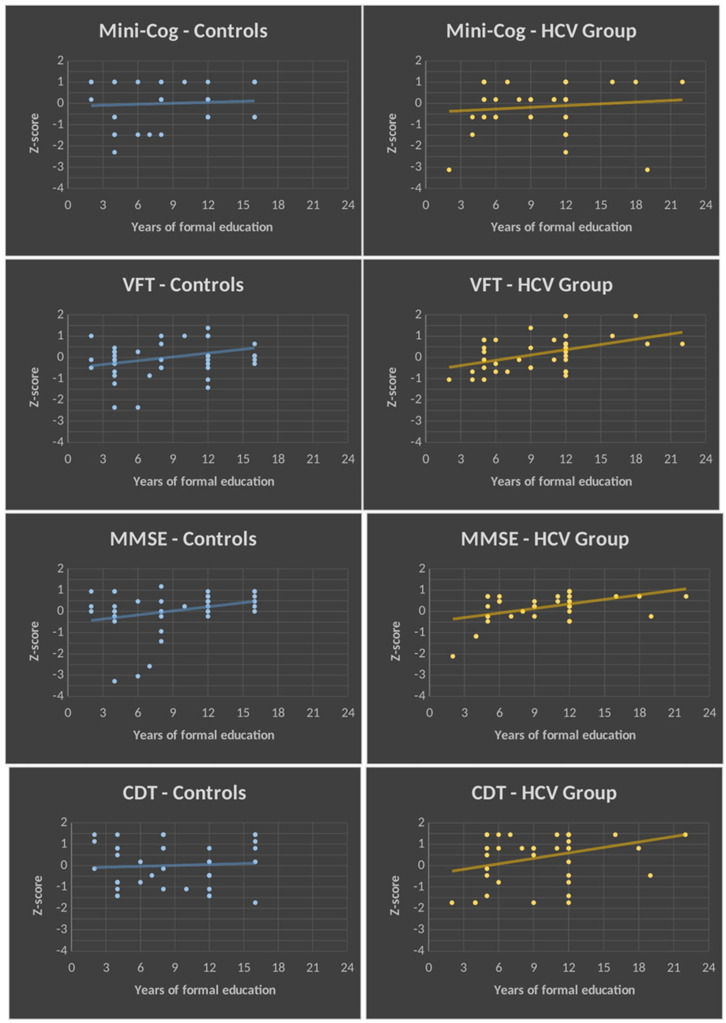
Regression lines showing the relationship between cognitive performance and years of education. Regression lines between Z-scores of cognitive tests (dependent variables) are on the *y*-axis, and years of education (independent variables) are on the *x*-axis for each group (control and HCV groups are represented in blue and yellow, respectively). Note that there are no group differences in the slopes of the regression lines for the Mini-Cog, VFT, and MMSE. In contrast, for the CDT, the slope of the regression line is significantly higher in the HCV group as compared to the control group (*p* < 0.05). CDT, clock drawing test; MMSE, Mini Mental State Examination; VFT, verbal fluency test (category: animals); HCV, hepatitis C virus; *p*, proof value.

The triple interaction is explained by group differences in the slopes of the regression lines between performance (Z-scores) and years of education (Figure 2). For the Mini-Cog, the two groups did not differ, because in both cases years of education was not related to performance on this test. For the MMSE and VFT, an increase in performance was associated with an increase in the number of years of education. However, the rate of increase did not differ between the two groups. In contrast, for the CDT, the rate of improvement in performance with education was significantly higher in the HCV group as compared to controls.

### 3.5. Analyses of the Associations with the Viral Load in HCV Carriers

The association between viral load and educational level did not reach significance. Viral load was also not associated with cognitive performance in any of the four tests or with the two sexes. All correlation coefficients were <0.10 (*p* > 35% in all cases).

## 4. Discussion

We did not find differences in the performance on the four cognitive screening tests between the two groups (HCV carriers and controls). We found a highly significant effect of education on performance in both groups. However, the relationship between education and test performance was found to depend on the group and the type of cognitive test. There were no differences in the slopes of the regression lines in the Mini-Cog, MMSE, and VFT. Conversely, in the CDT, the rate between performance and education was found to be greater in the HCV group as compared to controls. Thus, a possible difference between the two groups in the CDT performance might be obscured by the greater improvement in cognitive performance provided by education in HCV carriers as compared to controls.

### 4.1. Absence of Group Differences in the Performance of General Screening Tests (First Main Objective)

The lack of a significant effect of HCV on cognitive performance in asymptomatic elderly people infected for more than 20 years can be interpreted considering at least four possible explanations: First, the presence of the virus in asymptomatic patients might not cause any long-term damage to the nervous system or may not reach a magnitude capable of causing any permanent cognitive impairment. Secondly, plastic mechanisms could be operating in the brains of infected individuals and counteract any possible deleterious effects of the virus on the nervous system. Thirdly, general screening instruments may lack adequate power to detect subtle cognitive changes. Fourthly, cognitive reserve could confer protection against cognitive decline in HCV carriers.

The first and second possible explanations are supported by previous studies, which did not find any cognitive impairment in HCV-infected patients without cirrhosis [44,45,46]. The third is corroborated by Forton et al., who described that subtle cognitive impairments in patients with mild HCV infection can be only detected by specific computerized tests [44,47,48]. The fourth is supported by previous findings reporting that cognitive reserve may protect HCV-infected patients [20,49,50]. Regarding the fourth possible explanation specifically, we analyzed the effect level of education on cognitive performance.

### 4.2. Effect of Level of Education (Second Main Objective)

The finding that the Mini-Cog was not influenced by educational level may reflect that this test is easy to perform, and both groups performed equally irrespective of their educational level. As there was no group difference in the Mini-Cog and we did not find any evidence of an effect of education on performance, we concluded that the Mini-Cog definitively did not discriminate any putative cognitive differences between HCV carriers and controls.

The MMSE and the VFT were strongly influenced by education, and any possible group differences in the effect of education should be analyzed considering the data on the interaction between group and education in these tests.

In the CDT, we noticed that the low-educated subgroup of HCV carriers performed worse than the corresponding low-educated control subgroup. However, an adequate study of group differences in the effect of education on performance for specific tests would require the verification of the interaction between group, education, and type of test.

### 4.3. Group Differences in the Effect of the Educational Level on Performance in the Different Types of Tests (Third Main Objective)

The significant effect of the triple interaction (group, education, and type of test) on cognitive performance indicated that the effect of education on performance did not operate at the same level for the four different tests across the two groups.

The analyses of group differences in the slopes of the regression lines between years of education and test performances revealed that the strength of the relationship depended on the group and the type of cognitive test. For the CDT, the slope of the regression line was greater in the HCV group as compared to the controls. For the MMSE and VFT, both groups showed a similar rate of increase in performance as their years of education increased. For the Mini-Cog, both groups did not show any significant increase in performance as their education level increased. Therefore, education conferred a significantly higher increase in the performance rates on the CDT in the HCV carriers as compared to the rate of increase observed in the controls.

The specific effect on CDT performance is consistent with the overlap between brain regions affected by the neurotropism of HCV and the brain regions involved with the performance on the CDT. Previous investigators have described the critical role of the frontoparietal network in the CDT task [51]. In addition, neuronal loss and neuroinflammation in frontal regions have been observed in HCV-infected individuals [9]. Neurotropism induced by HCV infection is found in the frontal cortices, cingulate gyri, medulla, and basal ganglia [10,52].

### 4.4. Viral Load: Education and Cognitive Performance (Secondary Objective)

Viral load was not associated with education level, sex, or cognitive performance. In the patients infected with HCV, the lack of association between cognitive performance and viral load indicated that cognitive performance was independent of liver dysfunction, as described by other authors [8,49,53]. In addition, the viral load was not associated with education level, indicating that education did not interfere with viral load.

The high viral load found in the infected group may reflect that the patients in the present study were waiting for the release of DAA agents according to the most recent protocol approved by the Brazilian health authorities [5,6].

### 4.5. Strengths

One strength of this study was the use of a sample that was clinically asymptomatic and had carried the virus for more than 10 years. Therefore, this sample is representative of chronic infection without clinical symptoms. This is also supported by the finding that the degree of fibrosis had a balanced distribution in elderly individuals with HCV. Furthermore, on the Child–Pugh prognostic scale, most individuals were rated A5 (clinically compensated), making it clear that hepatic dysfunction and the degree of fibrosis did not affect their performance.

The analysis of the viral characteristics of elderly individuals with HCV showed that the absolute majority had genotype 1—the most prevalent in Brazil and in the world—similar to other studies [5,6]. This finding suggests that the results described here can be generalized to other countries and are not restricted to the Brazilian sample [54,55]. HCV genotype 3 is associated with an increased risk of cirrhosis and hepatocellular cancer [56,57]. In the present study, only two patients had genotype 3. Although we could not conduct any statistical analyses, the average cognitive performance of these two patients was similar to that of the genotype 1 group.

Although most of the participants were female, we did not find any effect of sex. This finding supports the absence of a sex effect on cognitive performance [18]. It should be mentioned that the proposed effect of sex on cognitive performance is still a matter of controversy [17,18,19]. With respect to a sex effect on cognition, a previous study reported that the prevalence of dementia syndrome in females is almost double that in males [19]. However, despite the controversy regarding a sex effect on cognitive performance, our finding of a lack of sex differences strengthens the cognitive integrity of our sample.

In the pairing of the groups, we used factors that should always be considered in the interpretation of performance on the cognitive screening tests, such as age, sex, depression, comorbidities, sedentary lifestyle, and formal years of education, which have been shown in previous studies to influence performance on the tests and modify the cutoff points [47,48,58]. It should be stressed that the two groups had educational backgrounds from the same period and were similar to one another [59]. Although the matching process was not perfect, there were no statistically significant differences between the two groups regarding any of the factors. Additionally, it should be mentioned that the ANCOVAs were adjusted for age and sex. Therefore, it is unlikely that the results would be explained by any putative differences regarding these factors.

### 4.6. Limitations

One limitation of this study is the observational cross-sectional design. Moreover, the small total number of participants limits the understanding of cognitive changes in HCV, as well as the generalization of these data to the entire population. Indeed, if the sample size is too small, the data are not representative of the population that is being sampled and, consequently, the results cannot be confidently extrapolated for the whole population. Additionally, a small sample size increases the likelihood of a type II error and decreases the power of the study. However, in the present investigation, we adjusted the sample size based on the required confidence level and margin of error. Furthermore, the central limit theorem states that, regardless of the distribution of the population, the distribution of the sample’s mean approximates a normal distribution if the sample size is equal to or greater than 30. Thus, we considered that our sample size (n = 41) was adequate for an initial exploratory analysis. However, the question of sample size is particularly crucial in the low-educated subsamples. Further studies should be conducted using larger samples, particularly in low-educated subjects.

The tests used can also be considered a limitation, because they may not have the ability to evaluate the areas of the CNS affected by HCV, and their results may also be obscured by education level. These brief tests can lead to clinical misinterpretations, because many of them depend on a high demand for reading and writing skills. This fact may have also influenced the outcomes of this study [49,60,61,62].

Previous studies have argued that there is a rationale for using both the MMSE and the CDT when screening for cognitive impairment, as the MMSE measures mostly verbal skills and, thus, could miss patients with early executive dysfunctions [63]. Even though some of the tests used here share similar tasks, the combined use of these tests has been shown to increase the accuracy in the detection of mild cognitive impairment as compared to the isolated use of each one [64]. However, these cognitive tests are quick screening instruments, and there is a need for more in-depth testing. Future studies should be conducted using tasks that explore different cognitive functions, such as attention and executive function.

Here, the four neuropsychological tests were always administered in the same sequence. As the two groups performed the tests in the same order, any impact of the sequence of the tests was distributed evenly across the two groups. However, the impact of completing one test on the performance of the others should be considered in a future study. Counterbalanced designs would allow for isolating the main effects of HCV from order effects.

Increased inflammation is commonly reported in the aged population, which could potentially also contribute to cognitive impairment [65,66]. However, in the present study, we did not measure inflammatory markers. Therefore, we could not analyze the possible margin or probability that the study outcome was linked to inflammatory markers associated with HCV infection as compared to those of the aged population.

Another limitation is the fact that we did not have neuroimaging data. Thus, we cannot entirely exclude the possibility that some participants may have had a greater cerebrovascular lesion load than others. The number of subjects with cerebrovascular lesions was expected to be higher in the HCV group as compared to the controls. Among other risk factors, positive correlations were found between HCV and carotid atherosclerosis/cardiovascular disease [67]. In addition, HCV eradication has been associated with a lower risk of long-term cerebrovascular events [68]. For all of these reasons, neuroimaging data should be included in a future study.

Finally, in the context of HCV eradication, a previous study investigated disturbances in the brain’s bioelectrical activity in HCV-positive patients before and 24 weeks after interferon-free therapy with DAAs. These authors used visual and brainstem-evoked potentials and found an improvement in performance after DAA treatment [69]. DAAs also reduced neuroinflammation and cerebral edema [70]. These studies suggest that neurotropic processes are modifiable. In the present study, all HCV patients were tested free of any treatment. Thus, a future study should be conducted assessing the same patients after DAA treatment

## 5. Conclusions

The cognitive performance in general screening instruments of elderly people with or without HCV infection did not differ. Educational level influenced performance in both groups. In the CDT, higher education gave further specific protection in elderly people with HCV. Thus, possible group differences in the CDT performance might be obscured by the educational level. Future studies should be performed using specific tests rather than general screening tests such as the Mini Mental State Examination, Mini-Cog, or verbal fluency. The selected tests should be associated with the brain regions that are known to be affected by HCV. Additionally, our data also indicate that the choice of the adequate tests to evaluate cognitive performance in HCV-infected subjects should not include those that use items that are heavily influenced by formal education. Finally, the importance of finding adequate tests to assess early subtle cognitive deficits in asymptomatic elderly people with HCV may be useful to provide precocious therapeutic interventions in these patients.

## Figures and Tables

**Table 1:** Clinical, epidemiological, and virological characteristics of the HCV group (*n* = 41).

Variables	Description of the Variables	Values
Route of transmission	Transfusion	30 (73.1%)
	Do not know	7 (17.1%)
	Sexual route	2 (4.9%)
	Needles	2 (4.9%)
Year of exposure (year)	Range	1968–1995
Year of the serological test *	Range	1995–2017
HCV genotype	Genotype 1	39 (95.1%)
	Genotype 3	2 (4.9%)
Viral load Child–Pugh scale	Mean A5	2,617,700 UI/mL
		39 (95.1%)
	>A5	2 (4.9%)
Degree of liver fibrosis	F0-F1	15 (36.6%)
	F2	14 (34.1%)
	F3	5 (12.2%)
	F4	7 (17.1%)

HCV—hepatitis C virus; IU—international units; mL-milliliters; A5—Child–Pugh score 5 classification; Metavir scores for fibrosis grading: F0 (absence of fibrosis), F1 (minimal fibrosis), F2 (septal fibrosis), F3 (various portal-portal septal fibrosis or fibrosis without cirrhosis), and F4 (fibrosis–cirrhosis). * The first test to identify the antibody anti-HCV was developed in 1992 [5].

**Table 2:** Demographic and clinical variables of the two groups (control and HCV groups).

Variables	Subdivisions of the Variables	Total	HCV	Controls	*p*-Value (HCV vs. Controls)
Participants (%)		82 (100%)	41 (50%)	41 (50%)	
Education	High	51 (62.2%)	27 (65.9%)	24 (58.5%)	*p* = 0.47
	Low	31 (37.8%)	14 (34.1%)	17 (41.5%)	
Years of formal education	Mean (standard deviation)	9.24 (4.49)	9.83 (4.38)	8.66 (4.58)	*p* = 0.24
Age	60 to 69 years old	46 (56.1%)	32 (78%)	14 (34.2%)	*p* = 0.40
	70 to 79 years old	29 (35.4%)	7 (17.1%)	22 (53.6%)	
	80 to 89 years old	7 (8.5%)	2 (4.9%)	5 (12.2%)	
Sex	Number of females (%)	63 (76.8%)	28 (68.3%)	35 (85.4%)	*p* = 0.07
Arterial hypertension	Presence	56 (68.3%)	24 (58.5%)	32 (78%)	*p* = 0.10
Type 2 diabetes	Presence	27 (32.9%)	14 (34.1%)	13 (31.7%)	*p* = 0.82
Sedentary lifestyle	Presence	61 (74.4%)	29 (70.7%)	32 (78%)	*p* = 0.61

HCV—hepatitis C virus; high education: 8 or more years of formal education; low education: less than 8 years of formal education. All participants reported the absence of traumatic brain injury with loss of conscience. All participants had normal glucose levels and normal tension blood pressures at the moment of the assessment; *p*, proof value. Note that the two groups did not differ in any of the abovementioned variables.

**Table 3:** Cognitive performance of 41 uninfected controls and 41 asymptomatic HCV carriers (raw scores).

Cognitive Tests	Total Participants (82)	HCV Group (41)	Controls (41)
Mini-cog	3.68 (1.275)	3.59 (1.34)	3.78 (1.21)
MMSE	26.41 (3.59)	26.83 (2.75)	26.00 (4.26)
CDT	6.10 (3.43)	6.73 (3.63)	5.46 (3.14)
VFT	15.09 (4.82)	15.56 (4.25)	14.61 (5.35)

CDT—clock drawing test; MMSE—Mini Mental State examination; VFT—verbal fluency test (category = animals); HCV—hepatitis C virus. Each value is reported as the mean (standard deviation). All of the comparisons between HCV and controls failed to reach significance.

## Data Availability

The data supporting this research is available upon request to the corresponding author. We also declare that no new data were created.
